# Investigating LSA - a ‘legal high’ analogue to LSD, frequently used in the digital realm with relatively unknown effects

**DOI:** 10.1192/j.eurpsy.2024.1150

**Published:** 2024-08-27

**Authors:** P. Castro, J. Maia

**Affiliations:** FMABC, Santo André, Brazil

## Abstract

**Introduction:**

Psychedelics are substances consumed for recreational use, the majority of these psychoactive substances are illegal and hard to obtain. Therefore, there is a demand for psychedelics legal and easier to access, these types of drugs are called ‘legal highs’. LSA (lysergic acid amide) is one of these new psychoactive substances, this drug is searched because it is known to have an effect similar to LSD. LSA has negative effects on body functioning not fully understood by the medical field.

**Objectives:**

This project aims to conduct a systematic review of the scientific health literature on LSA.

**Methods:**

The following information was included in this review: articles reporting original data on physical effects, neurobiological effects, various bodily symptoms, social and cultural aspects in humans related to LSA, published in English, Portuguese, Spanish, Italian, and French. Studies involving animals, in vitro research, botanical studies, and non-original research were excluded. The following keywords were searched in the PubMed, Google Scholar, and Web of Science databases: (ergine or d-lysergic acid amide or LSA or d-lysergamide or lysergic acid amide). This study followed the PRISMA statement for systematic reviews and PRISMA checklist. The resulting data were tabulated and analyzed according to relevance.

**Results:**

LSA, also known as ergine, is an ergot alkaloid with a chemical formula very similar to LSD. Ergine is found in plants of the Convolvulaceae family and is primarily consumed through chewing the seeds of these plants, soaking them in alcohol, or preparing an extract. The amount of LSA in each seed is inconsistent, making it unpredictable how much will be consumed, and these seeds may contain other harmful compounds.

LSA is a partial agonist and antagonist of serotonergic receptors, with a preference for 5-HT1A and 5-HT2, and stimulation of D2 is related to nausea. It can cause symptoms including euphoria, hallucinations, anxiety, nausea, weakness, fatigue, tremors, and elevated blood pressure. In some cases, the use of LSA is associated with the use of other drugs, and there are case reports of LSA-induced PRES (Posterior Reversible Encephalopathy Syndrome), post-use suicides and the need for hospitalization due to psychosis-like states.

Studies conducted on the quality of information about LSA on digital platforms indicate misinformation with incorrect data that can be harmful to those who ingest the drug. Additionally, there are studies suggesting that LSA may improve symptoms of cluster headaches.

**Conclusions:**

LSA is a legal drug in most countries, with widespread misinformation on the internet and limited control over its use. There are potential serious adverse effects caused by the drug, and it is often associated with other psychoactive substances. Greater knowledge about the drug is needed for diagnosing its use and abuse, as well as for educating the public.

**Disclosure of Interest:**

None Declared

